# Ion mobility spectrometry combined with ultra performance liquid chromatography/mass spectrometry for metabolic phenotyping of urine: Effects of column length, gradient duration and ion mobility spectrometry on metabolite detection

**DOI:** 10.1016/j.aca.2017.06.020

**Published:** 2017-08-22

**Authors:** Paul D. Rainville, Ian D. Wilson, Jeremy K. Nicholson, Giorgis Issacs, Lauren Mullin, James I. Langridge, Robert S. Plumb

**Affiliations:** a Waters Corporation, Milford, MA, 01757, USA; b Biomolecular Medicine, Division of Computational and Systems Medicine, Department of Surgery and Cancer, Imperial College London, Sir Alexander Fleming Building, Exhibition Road, South Kensington, London SW7 2AZ, UK; c MRC-NIHR National Phenome Centre, Department of Surgery and Cancer, Imperial College London, IRDB Building, Du Cane Road, London W12 0NN, UK

**Keywords:** Metabonomics, Metabolomics, Metabotyping, Mass spectrometry, Ion mobility spectrometry

## Abstract

The need for rapid and efficient high throughput metabolic phenotyping (metabotyping) in metabolomic/metabonomic studies often requires compromises to be made between analytical speed and metabolome coverage. Here the effect of column length (150, 75 and 30 mm) and gradient duration (15, 7.5 and 3 min respectively) on the number of features detected when untargeted metabolic profiling of human urine using reversed-phase gradient ultra performance chromatography with, and without, ion mobility spectrometry, has been examined. As would be expected, reducing column length from 150 to 30 mm, and gradient duration, from 15 to 3 min, resulted in a reduction in peak capacity from 311 to 63 and a similar reduction in the number of features detected from over ca. 16,000 to ca. 6500. Under the same chromatographic conditions employing UPLC/IMS/MS to provide an additional orthogonal separation resulted in an increase in the number of MS features detected to nearly 20,000 and ca. 7500 for the 150 mm and the 30 mm columns respectively. Based on this limited study the potential of LC/IMS/MS as a tool for improving throughput and increasing metabolome coverage clearly merits further in depth study.

## Highlights

• Ion mobility spectrometry (IMS) significantly increased the number of analytes detected during the LC-MS of urine.
• Nearly ca. 20,000 features were seen for urine using LC-IMS-MS in a 15 min analysis compared to ca. 16,000 by LC-MS alone.
• In a 3 min analysis using a 30 mm column nearly 7600 features were detected with combined IMS and MS.
• For high throughput analysis a 75 mm column and a 3 min analysis was a good compromise between speed and features detected.
• The use of IMS also improved the quality of the mass spectra obtained.

## Graphical abstract

**Figure:**
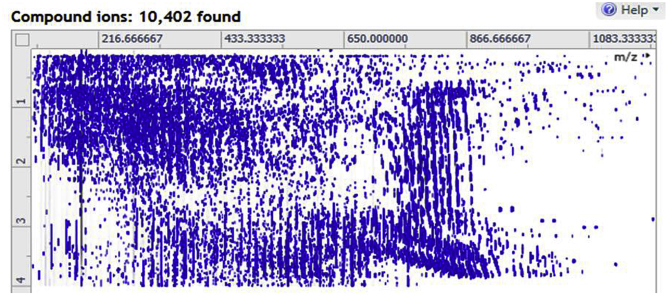

## 1 Introduction

The use of metabolic phenotyping (metabonomics/metabolomics) to discover biomarkers of organismal response to environmental and physiological change is now widespread. In biomedical applications metabolic phenotyping, or metabotyping [1], [2], is being deployed as a method for finding novel, mechanistic, biomarkers of disease with obvious potential for improving diagnosis, patient stratification and both predicting and monitoring patient response to therapy. Because of the need for the robust identification of analytes current analytical platforms for metabolic phenotyping are based around techniques such as nuclear magnetic resonance (NMR) spectroscopy or mass spectrometry (MS), which have the potential for structural characterization as well as detection and quantification. In the case of MS analysis can be performed by direct infusion (DIMS) [3] or, following hyphenation, in combination with a separation technique. Separation techniques such as GC-MS [4], LC-MS [5], [6], SFC-MS [7] and CE-MS [8] have all been employed, to greater or lesser extents, for the metabotyping of a very wide range of clinical samples, from biofluids such as urine, blood-derived products, bile etc., to cells, tissue and faecal extracts etc. LC/MS-based methods, particularly those centred on its more efficient ultra performance, or ultra high performance variants (UPLC/UHPLC), based on separations made using high flow rates and sub 2 μm packing materials, have delivered improved methods for metabolic phenotyping compared to conventional HPLC-MS approaches. However, there remains the difficulty of balancing the desire for rapid, high throughput, analysis versus the need to maximise metabolome coverage for biomarker discovery. In particular, as the separation time is reduced to increase sample throughput ion suppression (due to peak co-elution) increases, reducing the number of features detected (e.g. see Refs. [9], [10]). Another reason for the loss of some of these features can be that the signals arising from low intensity analytes, which might well be detected with longer separations, are effectively lost due to both “system noise” and co-elution with ions of much higher intensity. Indeed, even when separations are not compressed to maximise throughput, the loss of signals for some low intensity analytes co-eluting with higher intensity ones is likely. An obvious way of reducing the problems of co-elution is to use strategies such as 2-dimensional separations but this clearly does not solve the problem of maximising throughput. Another potential means of maximising metabolite detection without increasing analysis time is to employ ion mobility spectrometry (IMS) prior to MS detection in a hyphenated LC-IMS-MS system. The use of IMS also offers the potential to gain further structural information via accessing the collision cross section information for molecules of interest. The rapid time scale of ion mobility separations, typically in the 10s of milliseconds range, makes such a configuration ideal for coupling between UPLC-based separations, with peaks eluting over a few seconds and TOF mass spectrometry which operates on a microsecond time scale. The use of the “drift time” within the ion optics can allow analytes of interest to be separated and detected even in the presence of a co-eluting isobaric species. This orthogonal separation therefore provides an increase in peak capacity, in an analogous manner to two-dimensional LC, but without an increase in analysis time. However, it is important to recognize that, as this separation is performed post ionization in the vacuum region of the mass spectrometer, whilst potentially increasing the number of analytes detected using IMS will not compensate for the loss of analytes as a result of ion suppression as this is an ionization phenomenon.

Whilst LC-IMS has been widely employed in areas such as protein characterization and proteomics [11], the analysis of pharmaceuticals [12] and drug metabolite identification [13] etc., there have to date been relatively few reports of direct applications of IMS-MS [14], [15], [16], [17], [18] or LC-IMS-MS to metabolic phenotyping [19], [20], [21] or lipidomics [22], [23], [24] despite the obvious potential of the technique. Here we describe the results of the investigation of the effect of integrating IMS with gradient reversed-phase UPLC, using different column lengths and gradient durations, as a means of enhancing the data obtained for the metabotyping of human urine.

## 2 Experimental

### 2.1 Chemicals

Formic acid, methanol and acetonitrile (ACN) were purchased from Thermo Fisher Scientific (Pittsburgh, PA, USA). Water was obtained from an in-house milli Q filtration system Millipore (Billercia MA, USA).

### 2.2 Sample preparation

A number of 20 μl aliquots of midstream control human urine were obtained from 6 volunteers and stored frozen at −20 °C prior to analysis. The samples were analysed individually in triplicate and also as a pooled sample (prepared by mixing equal aliquots of urine from all of the volunteers). Following thawing the samples were diluted with 80 μl of pure water and centrifuged at 13,000 rcf for 5 min at 4 °C after which 80 μl was taken and transferred to maximum recovery glass vials for analysis.

### 2.3 Chromatography

The separations were performed on either a 2.1 × 150 mm, 2.1 × 75 mm or 2.1 mm × 30 mm ACQUITY UPLC 1.8 μm HSS T3 columns using an ACQUITY Ultra Performance LC chromatography™ System (Waters^®^ Corporation, MA, USA). Columns were maintained at 40 °C and eluted with acetonitrile – aqueous formic acid (0.1% v/v) using a series of linear gradients over either 15, 7.5 or 3 min (depending on column length) at 600 μL/min starting at 2% ACN and, after a hold of either 1 min, 0.5min or 0.2 mins at this composition, rising first to 15, then 50 and finally to 95% ACN at the end gradient (for column/run-specific details see Table 1). The acetonitrile composition was then held at 95% ACN for 1 min before returning to the initial conditions for column re-equilibration prior to the next analysis. These conditions ensured that the number of column volumes defining the gradient remained constant for all column lengths. The column eluent was directed to the mass spectrometer for analysis. In addition to these conditions an experiment was performed whereby the 2.1 × 75 mm column was eluted with an accelerated gradient utilizing a flow rate of 800 μL/min.

**Table 1:** Gradient conditions utilized during experiments on 150, 75 and 30 mm length columns.

Gradient Composition %B	30 mm min	75 mm Rapid Gradient min	75 mm min	150 mm min
2	0–0.2	0–0.3	0–0.5	0–1.0
2–15	0.2–0.8	0.3–0.9	0.5–2.0	1.0–4.0
15–50	0.8–2.2	0.9–2.2	2.0–4.5	4.0–9.0
50–95	2.2–3.0	2.2–3.0	4.4–7.5	9.0–15
2	4.0	4.0	8.5	16.0

### 2.4 Mass and ion mobility spectrometry

Mass spectrometry was performed on a Waters Synapt G2-Si (Waters Corporation, Wilmslow, UK) operated in positive ion mode with the resolving quadrupole set to a wide pass mode, the collision cell was set to alternate between a collision energy of 5eVand 25eV every 50msec with a 5msec inter scan delay. The instrument was operated with in electrospray mode with Lockspray enabled. The capillary voltage was set to 2.5 kV, the cone voltage 30 V, the source temperature 120 °C, the nebulizer was operated gas temperature was 600 °C at a flow rate of and 1200 L/hr. The data was collected in either Data Independent Acquisition (DIA) MS/MS mode or ion mobility enabled DIA mode. The ion mobility cell was filled with a helium cell gas flow of 180 mL/min and a voltage of 2 V applied between the source and end plate using a flow of 90 mL/min, a wave velocity of 600 m/s a wave height of 40 V and an EDC delay coefficient of 1.58 V. Leucine enkephalin was employed as the lockspray solution at a concentration of 50fmol/μL with a frequency of 11 with a scans to average set to 5. The raw data obtained was processed using Progenisis QI (NonLinear Dynamics, Newcastle, UK) using medium sensitivity peak detection settings, the derived peak/intensity lists were analysed using EZInfo (Umetrics, Umea, Sweden). The peak picking parameters employed in this study included the removal of both isotopes and adducts from the LC/MS data. The software also facilitated the detection of ions separated in the ion mobility domain.

## 3 Results and discussion

### 3.1 Effects of column length and gradient duration on feature detection in UPLC-MS

The optimal operation of sub 2-μm LC/MS for small molecule analysis, in gradient elution mode, requires analytical flow rates in the range 0.5–0.9 mL/min (for a 2.1 mm diameter column), which results in peaks of widths varying between 1.5 and 3 s. To evaluate the effect of column length on the number of features detected the analysis of control human urine samples obtained from 6 volunteers was analysed, both individually (in triplicate) and as a pooled sample (for the purposes of this study a detected feature is defined as a discrete, unique LC/MS peak separated by either, retention time, *m*/*z* or ion mobility drift time). In this study positive ion ESI MS was selected as the mode of detection only as, although negative ion LC/MS does enable the detection of some analytes that are not detected using positive ion MS, in our experience positive ESI analysis of urine results in the detection of a greater number of ions. Thus detection using positive ESI creates a much higher “peak density” than negative ESI for the same sample, and this represents a greater analytical challenge when attempting to maximise metabolome coverage.

**Fig. 1:**
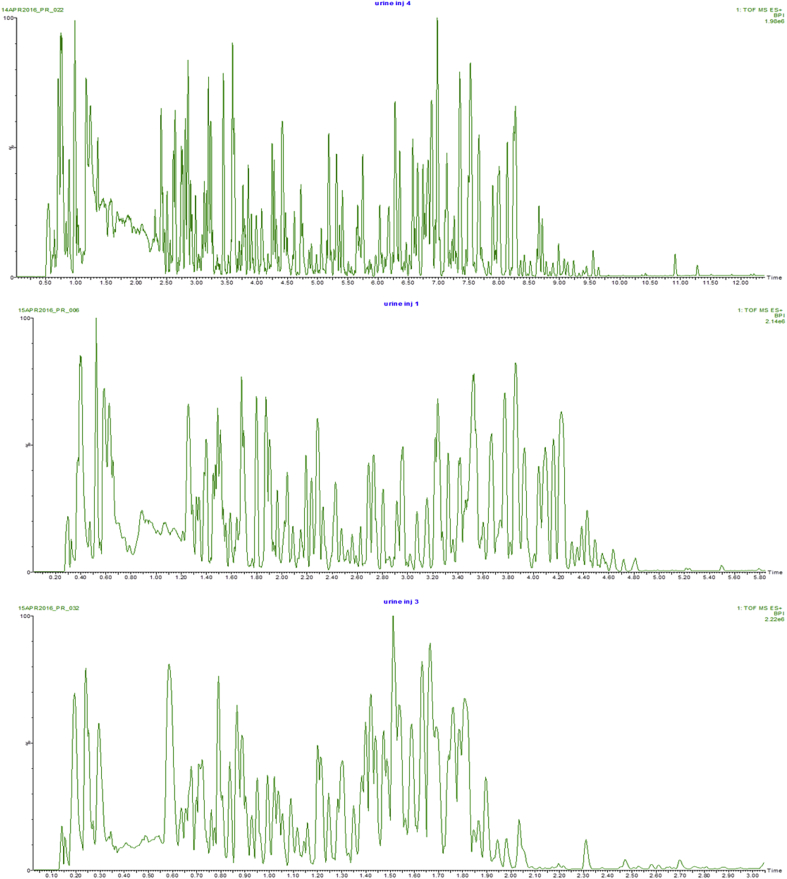
Analysis of human urine using gradient durations of 15, 7.5 or 3 min and column lengths of 150, 75 or 30 mm. The top chromatogram shows the BPI trace obtained from the UPLC-MS analysis of human urine using a 2.1 × 150 mm column and a gradient duration of 15 min, the centre chromatogram shows the same sample analysed using a 2.1 × 75 mm column and a gradient duration of 7.5 min, the lower chromatogram shows the same sample analysed using a 2.1 × 30 mm column and a gradient duration of 3 min.

**Fig. 2:**
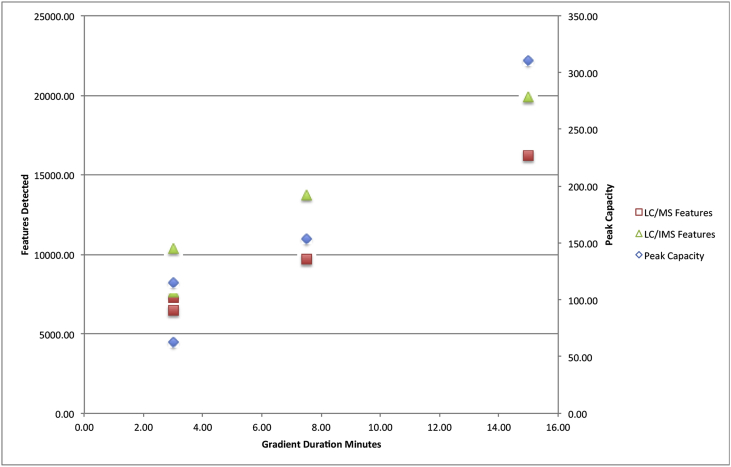
The relationship between gradient duration, peak capacity and the number of mass spectrometric features (ions) detected using columns of 30, 75 or 150 mm in length and either UPLC/MS or UPLC/IMS/MS (constructed from the data presented in Table 2).

The chromatographic analysis of human urine using a 15 cm length column and eluted with a linear solvent gradient from 5 to 60% acetonitrile over 15 min (at a flow rate of 600 μL/min) enabled the detection of in excess of 16,000 features (Table 2) in positive ion electrospray ionization with Data Independent Acquisition (DIA) ESI mode (using medium peak detection settings). This separation is shown in Fig. 1 (top), where it can be seen from the base peak ion (BPI) chromatogram that, following the initial hold on the gradient, the component peaks were well distributed across the first 10 min of the analysis. The relationship between the chromatographic conditions and the number of features detected in these urine samples with respect to column length and analysis time was then investigated by analysing them using 150, 75 and 30 mm long columns with the same solvents but with elution times of 15, 7.5 and 3-min duration respectively (Table 1 and Figs. 2 and S1). The use of these chromatographic conditions meant that the separations were scaled directly so as to ensure that the number of column volumes defining the gradient, in this instance 34, remained constant for each column. The peak capacity of the separations thus ranged from 311 (average peak width 2.9 s) when using the 150 mm column and its corresponding gradient elution over 15-min to 154 (average peak width 2.9 s) with the 75 mm column/7.5 min gradient and 85 with the 30 mm column/3 min (average peak width 2.0 s) gradient. The resulting BPI chromatograms obtained are shown in Fig. 1 and reveal LC peak widths varying from 3 to 1.5 s as the separation moves from the 150 mm column and 15-min gradient to the much faster 30 mm and 3-min gradient. As would be expected, the number of features detected as being present in the urine samples varied depending on the length of the column and corresponding analysis time (Table 1, Fig. 2). Thus from the maximum observed number of approximately 16,000 features detected for the longer 15-min separation performed on the 150 mm length column the number fell to 9600 with the 75 mm column and 7.5-min gradient and almost 8000 in the case of the 30 mm column and 3-min separation (Table 2, Fig. 1 and S1). These data show that, despite reducing the column length and gradient duration, it was still possible to obtain feature-rich analysis in a half or one third of the time of the 15 min analysis, thus offering opportunities for improving throughput. These finding are consistent with previous reports (e.g., [10], [25]).

**Table 2:** The effect of column length, gradient length and IMS on the number of features detected for the analysis of human urine using UPLC/MS and UPLC/IMS/MS.

Column Length (mm)	Gradient (min)	Flow Rate (mL/min)	Peak Capacity	Number of Features Detected UPLC/MS (% of 15 cm result)	Number of Features Detected UPLC/IMS/MS (% of non IMS 15 cm result)
150	15.0	0.6	311	16,192 (100%)	19,893 (118%)
75	7.5	0.6	154	9595 (59%)	13,701 (85%)
75	3.0	0.9	115	7267 (44%)	10,402 (64%)
30	3.0	0.6	63	6458 (39%)	7596 (47%)

Reducing the gradient duration on the 75 mm column to 3 min whilst increasing the flow rate to 900 μL/min resulted in the detection of approximately 7300 features. It is interesting to note that the combination of a 3-min gradient and a 75 mm column results in the detection of a similar number of features to the 30 mm column under the same conditions (7267 compared to 6468 respectively). This result indicates that, under the elevated flow rate conditions employed here, the number of features detected does not significantly increase proportionally to column length.

### 3.2 Effect of ion mobility spectrometry on peak detection

As indicated in the introduction, the use of IMS integrated into the UPLC-MS system offers the opportunity to provide an extra dimension of separation to the analysis process allowing for the resolution of co-eluting analytes based on their ion mobility. In this study we employed a Traveling Wave (TW) Ion Guide equipped mass spectrometer to effect the ion-mobility separation as previously described by Paglia *et a*l [21], [22], [24]. In TWIM-MS, an ion-mobility separation stage consisting of a stacked-ring ion guide with RF confinement is filled with an inert gas such as nitrogen or helium. Ions travel through the gas toward the MS detector propelled in an axial direction by a traveling-wave, DC voltage. Ions are thus separated in the gas phase according to their mobility through the gas, which is related to the charge, shape, and size of the ion. The time required for an ion to traverse the ion-mobility separation cell is called the drift time. From the drift time values, it is possible to derive the rotationally averaged collision cross section (CCS), which represents the effective area for the interaction between an individual ion and the neutral gas through which it is traveling. Thus, in addition to accurate mass data, including the ion mobility-derived CCS data also provides an additional physicochemical measurement that can be used for the annotation of metabolites and lipids as an aid to their identification (see Refs. [21], [22], [24]).

**Fig. 3:**
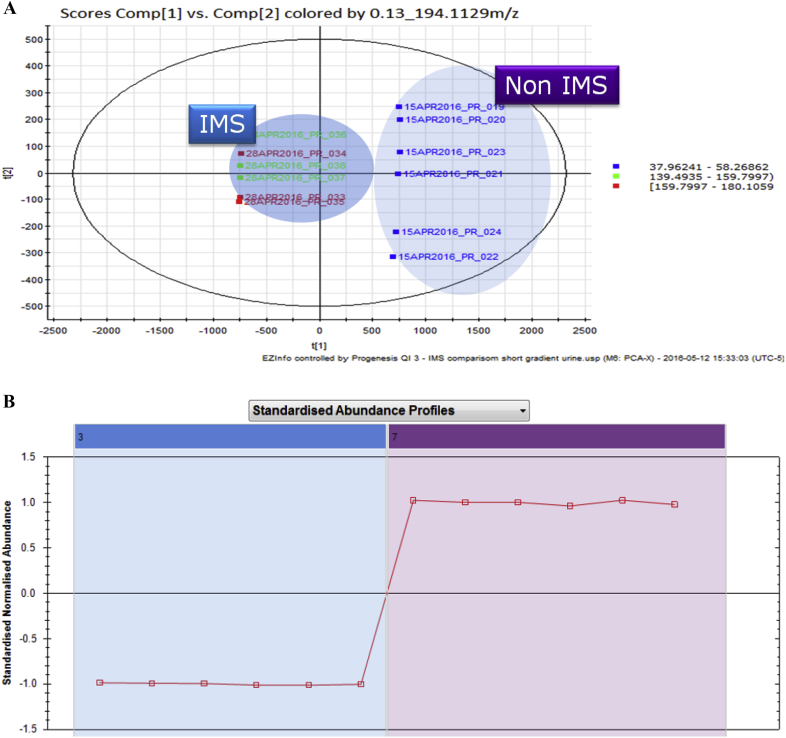
3a: Principal Components Analysis (Pc1/Pc2) of data obtained from replicate (n = 6) analysis of human urine using a 7.5 min UPLC/MS and UPLC LC/IMS/MS analysis on a 75 mm column. 3b: Standard abundance plot derived from data for the ion putatively ascribed to 4,8-dimethylnonanoyl carnitine obtained using conventional UPLC/MS and UPLC/IMSIMS analysis of human urine.

The chromatographic analysis of human urine described above was therefore repeated with IMS enabled before the collision cell in the mass spectrometer with the data obtained summarized in Table 2 and Fig. 3. The LC/IMS/MS analysis produced between a 25 and 40% increase in the number features detected in the urine analysis compared to UPLC/MS alone. The data acquisition rate was not affected by the IMS analysis with 9 data points acquired across a 2.5 s wide peak. Thus, the 15-min separation on the 150 mm column, when performed with IMS, yielded nearly 20,000 features compared to the ca. 16,000 without IMS. Similarly, the 7.5-min analysis combined with the 75 mm column resulted in an increase in the number of features detected from ca. 10,000 to nearly 14,000 whilst the 3 mm column, 3-min separation showed an increase in features from ca. 6500 to ca. 7600 (Table 2). A Two-dimensional peak density map (PDM) illustrating these results is shown in Fig. S2. In addition, the when IMS/MS was performed on a 75 mm column with a 3-min analysis 10,402 features were detected compared to 7267 without IMS, which represents 62.5% of the number of features detected using the 150mm/15-min analysis in just 20% of the analysis time. These data would suggest that the coupling of 7.5 cm column and the accelerated mobile phase linear velocities gives the best combination of throughput and feature detection. This could be rationalized by the fact that the 7.5 cm column delivers the best combination of chromatographic resolving power per unit time thus minimizing ion suppression, allowing the IMS/MS to isolate and detect the biological analytes.

**Fig. 4:**
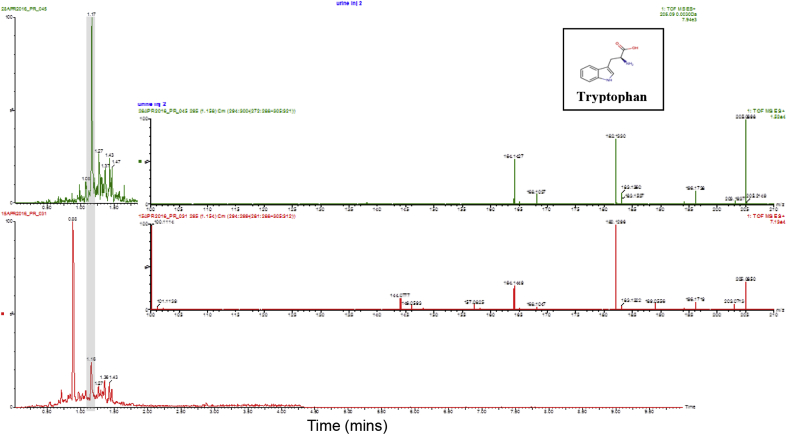
Extracted ion chromatograms and mass spectra for urinary tryptophan from the LC/MS analysis of pooled urine employing a 2.1 × 30 mm column and a 3 min gradient. The upper chromatogram/mass spectrum shows the IMS enabled separation and the lower chromatogram/mass spectrum the DIA data. The chromatographic peak for tryptophan (structure in inset) is highlighted by the vertical grey bar in both mass chromatograms.

Six replicate analysis of the urine samples were performed in both ion mobility and non-ion mobility mode using the 7.5 cm column and 7.5-min analysis methodology. The data derived from the UPLC/IMS/MS and conventional UPLC/MS analyses were then subjected to peak detection followed by multivariate statistical analysis. A simple principal components analysis showed that the IMS and non-IMS data were easily separated in PC1 and PC2 as shown in Fig. 3a. The higher variability observed in the non IMS analysis is due to the greater sensitivity of the non-IMS approach and hence the inclusion of compounds at the limit of analyte detection and increased noise, these were not observed in the IMS data. These data were also subjected to OPLS-DA analysis and the resulting loadings S-Plot (not shown) was used to identify the components unique to the IMS and non-IMS data. One of these ions, was tentatively identified (from a search of the Human Metabolome Database [26]) as potentially being due to the presence of 4,8-dimethylnonanoyl carnitine in the sample (mass spectrum provided in Fig. S3). A standardized abundance profile of the results for this ion is shown in Fig. 3b. It is perhaps noteworthy that, as well as only being detected in the IMS data, some ions were only detected in the conventional, non-IMS, data. The non-IMS-detected ions were however, of very low abundance in the UPLC/MS data and were probably not seen when IMS was used as a result of the reduction in sensitivity observed in IMS mode. The IMS-derived data was also mined for several well-known endogenous urinary metabolites including hippuric acid, pantothenic acid, tryptophan, indoxyl sulphate, phenylanaline, taurocholic acid, deoxycholic acid, and taurolithocholic acid. In general the DIA analysis data showed higher signal intensity than the IMS-derived data. The IMS data however, typically showed superior simplified ion chromatograms and MS spectra. An example of this is shown in Fig. 4 for the analysis of urinary tryptophan. As can be seen from the IMS chromatogram shown in Fig. 4, the IMS derived, upper, chromatogram shows a discrete peak for tryptophan at *m*/*z* = 205.090 whereas the DIA chromatogram shows the presence of several extra peaks. The tryptophan peak eluted with a retention time of 1.7 min and is clearly visible in the IMS data whilst only a small peak in the DIA data. The other peaks present in the DIA results were also detected in the IMS analysis but were clearly resolved from the tryptophan peak. The mass spectra of tryptophan obtained via DIA alone or with IMS shown with the mass chromatograms in Fig. 4 also demonstrate the improved data that can be obtained using this combination. In general we noted that performing IMS resulted in a loss in sensitivity of ca. 4-7-times compared to the DIA analysis, but the provision of “de-cluttered” MS data should improve spectral interpretation and database searching.

The results obtained in these studies indicated that by employing ion mobility in the mass spectrometry process prior to the time-of-flight tube the number of features detected was increased by 23, 41 and 17% for the 15, 7.5 and 3 min separations on the 15, 7.5 and 3 cm columns respectively. However, it is important to be clear that remarkable increases in features detected cannot be attributed to factors such as e.g. reduced ion suppression. Clearly, as ion suppression occurs in the ESI source, before the IMS cell, both IMS and non-IMS modes of data acquisition will be subject to the same level of ion suppression. The extra features resulting from the use of IMS also do not result from an enhancement in sensitivity as our experience shows that the IMS-TOF data for the analysis of pure standards shows a reduction in signal response compared to TOF MS alone. We speculate that the observed increase in features detected is most likely attributable to the separation of co-eluting compounds in the drift cell. As well as the resolution of co-eluting compounds during the ion mobility process the observed increase in features detected may also have been due to a number other factors, such as e.g., the co-elution of compounds which were isobaric (or having virtually identical *m*/*z* values), or were of low intensity and would therefore otherwise be lost in the “noise”, or that resulted from the separation of fragments derived from analyte ions. In addition, a further alternative rationalization of the increase in peak detection is, as a consequence of the extra dimension of selectivity provided by ion mobility cell, that the peak detection algorithms may be more able to differentiate between signals relating to a true peak and analytical noise. Comparison of the peak density maps for the LC/MS and LC-IMS-MS data shown in Figs. S1 and S2 clearly indicates that the UPLC/IMS/MS data contained significantly less noise compared to those for the conventional UPLC/MS analysis This suggests that a significant reason for the greater number of detected peaks is due to the reduction in extraneous noise and hence easier detection of “true” analyte peaks.

## 4 Conclusions

As shown here, the incorporation of IMS as a separation modality between LC and MS significantly increased the number of features detected in a metabolic phenotyping experiment. The increase in the number of features detected varied from 41% for the 7.5-min analysis to 17% for the 3-min analysis and 23% for the longer 15-min separation. The reason(s) for the observed increase in feature detection clearly needs further investigation but is most likely due to a combination of separation of co-eluting compounds, noise reduction, resolution of isobaric components and separation of fragment ions. Reducing the column length from 150 mm to 75 or 3 mm resulted in an almost linear reduction in feature detection. The addition of IMS to the 7.5 min analysis increased the number of features to 81% of the 15 min analysis and the 3 min analysis to almost 50% of the 15 min analysis.

The combination of reduced column lengths, gradient times and elevated flow rates combined with IMS has been shown to deliver greater than 60% of the number of features seen in the non-IMS data in just 20% of the analysis time of the conventional analysis (although the overlap in the identity of the ions detected in both modes needs further investigation). These data suggest that LC/IMS/MS combined with short columns and elevated mobile phase flow rates may be an attractive platform for the rapid analysis/screening of samples from large cohorts resulting from biobanking, epidemiological or large biomedical studies.
