# Supplementary material for: Ion mobility spectrometry combined with ultra performance liquid chromatography/mass spectrometry for metabolic phenotyping of urine: Effects of column length, gradient duration and ion mobility spectrometry on metabolite detection
Source: Anal Chim Acta. 2017 Aug 22;982:1–8. doi: 10.1016/j.aca.2017.06.020 (PMC5533171; doi:10.1016/j.aca.2017.06.020)
Supplement: Supplementary information [file mmc1.docx]

**Supplementary Information for**

**Ion Mobility Spectrometry (IMS) Combined With UPLC/MS For Metabolic Phenotyping of Urine: Effects of Column Length, Gradient Duration and IMS on Metabolite Detection.**

Paul D Rainville^1^, Ian D Wilson^2^*, Jeremy K Nicholson ^2,3^, Giorgis Issacs ^1^, Lauren Mullen^1^, James I Langridge^1^ and Robert S Plumb^1,2^*

^1^Waters Corporation, Milford, MA, 01757, USA.

^2^ Biomolecular Medicine. Division of Computational and Systems Medicine, Department of Surgery and Cancer, Imperial College London, Sir Alexander Fleming Building, Exhibition Road, South Kensington, London SW7 2AZ, UK.

^3^MRC-NIHR National Phenome Centre, Department of Surgery and Cancer, Imperial College London, IRDB Building, Du Cane Road, London W12 0NN, UK.

*Corresponding Authors. Email: [R.Plumb@imperial.ac.uk](mailto:R.Plumb@imperial.ac.uk) Tel: 00 44 207 594 3225

Email: [i.wilson@imperial.ac.uk](mailto:i.wilson@imperial.ac.uk) Tel: 00 44 207 5940730

**Figure S1.** Peak density maps for the UPLC/IMS/MS analysis of human urine. The top peak density map shows the result obtained using a 2.1 x 150mm column and a gradient duration of 15 minutes, the centre peak density map shows the same sample analysed using a 2.1 x 75mm column and a gradient duration of 7.5 minutes, the lower peak density map shows the same sample analysed using a 2.1 x 30mm column and a gradient duration of 3 minutes. Flow rate of 0.6 ml min for all experiments.

**Figure S2.** Peak density maps for the UPLC/IMS/MS analysis of human urine. The top peak density map shows the results obtained using a 2.1 x 150mm column and a gradient duration of 15 minutes with ion mobility separation enabled, the centre peak density map shows the same sample analysed using a 2.1 x 75mm column and a gradient duration of 7.5 minutes with ion mobility separation enabled, the lower peak density map shows the same sample analysed using a 2.1 x 30mm column and a gradient duration of 3 minutes with ion mobility separation enabled. Flow rate of 0.6 ml min for all experiments.


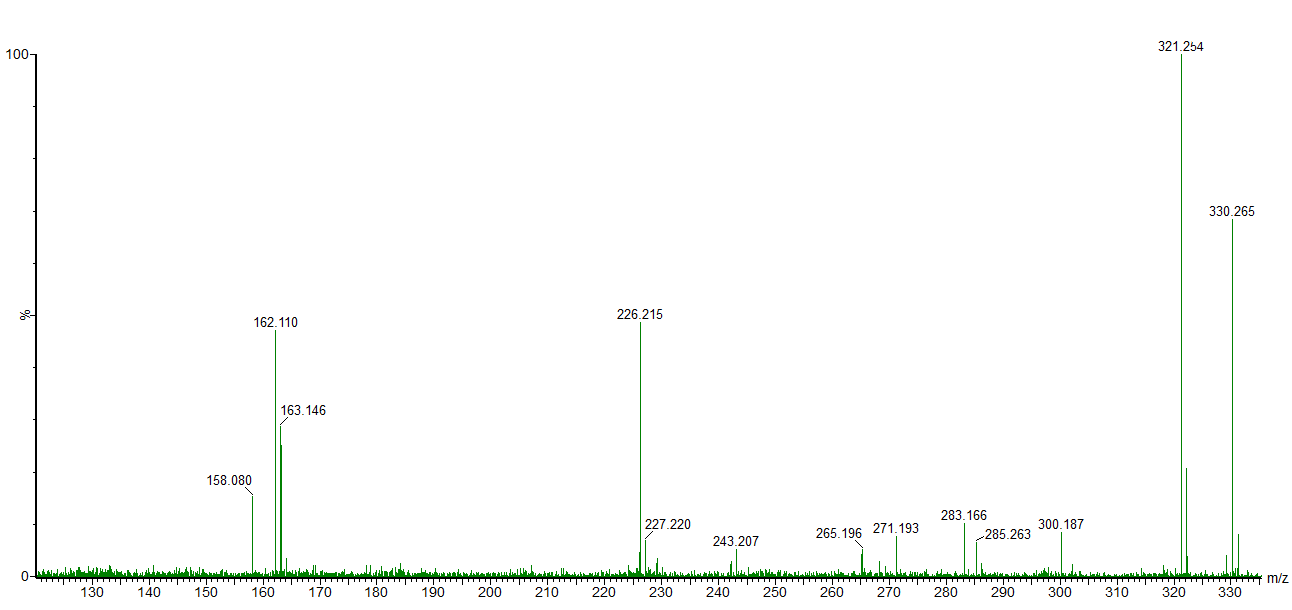


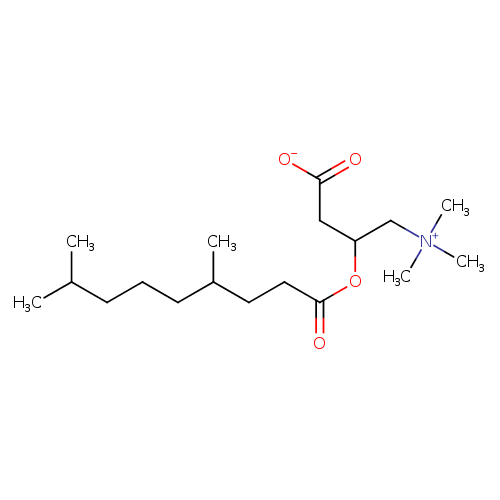


**4,8-dimethylnonanoyl carnitine**

**Figure S3.** UPLC-IMS-derived MS/MS spectrum of a peak tentatively identified as 4,8-dimethylnonanoyl carnitine based on data provided in the Human metabolome Database.
